# Supplementary material for: Relationship between Serum 25-Hydroxyvitamin D Level and Risk of Recurrent Stroke
Source: Nutrients. 2022 May 2;14(9):1908. doi: 10.3390/nu14091908 (PMC9099592; doi:10.3390/nu14091908)

Supplemental materials

List of contents:

Supplemental Table S1. Codes used for ascertainment of stroke events at baseline and during follow-up 3

Supplemental Figure S1. Flow diagram showing the selection of participants in this study 4

Supplemental Figure S2. Kaplan-Meier failure curve for incident recurrent stroke 6

Supplemental Figure S3. Kernel density estimate for probability density function of 25(OH)D stratified by participants with and without recurrent stroke 7

Supplemental Table S2. Sensitivity analysis results for the relationship between 25(OH)D and risk of recurrent ischemic stroke 8

Supplemental Figure S4. Restricted cubic splines showing 25(OH)D in relation to recurrent ischemic stroke with the lowest risk laying at 56.3 nmol/L 10

Supplemental Figure S5. Restricted cubic splines showing 25(OH)D in relation to recurrent hemorrhagic stroke with the potentially lowest risk laying at 41.2 nmol/L 11

Supplemental Figure S6. Restricted cubic splines showing 25(OH)D in relation to recurrent stroke in males with the lowest risk laying at 58.7 nmol/L 12

Supplemental Figure S7. Restricted cubic splines showing 25(OH)D in relation to recurrent stroke in females with the potentially lowest risk laying at 38.7 nmol/L 13

Supplemental Figure S8. Restricted cubic splines showing 25(OH)D in relation to recurrent stroke in patients less than 65 years with the lowest risk laying at 58.4 nmol/L 14

Supplemental Figure S9. Restricted cubic splines showing 25(OH)D in relation to recurrent stroke in patients no less than 65 years with the lowest risk laying at 53.3 nmol/L..15

Supplemental Table S1. Codes used for ascertainment of stroke events at baseline and during follow-up

Diseases	Source	Code
**Stroke**	HES (ICD 10)<br>HES (ICD 9)<br>Baseline (non-cancer illness code)	I60, I61, I62, I63, I64<br>430, 431, 432, 433, 434, 436<br>1081, 1086, 1491, 1583
**Ischemic stroke**	HES (ICD 10)<br>HES (ICD 9)	I63<br>433, 434
**Hemorrhagic stroke**	HES (ICD 10)<br>HES (ICD 9)	I60, I61, I62<br>430, 431, 432

HES = Hospital Episode Statistics; ICD = International Classification of Diseases

Supplemental Figure S1. Flow diagram showing the selection of participants in this study

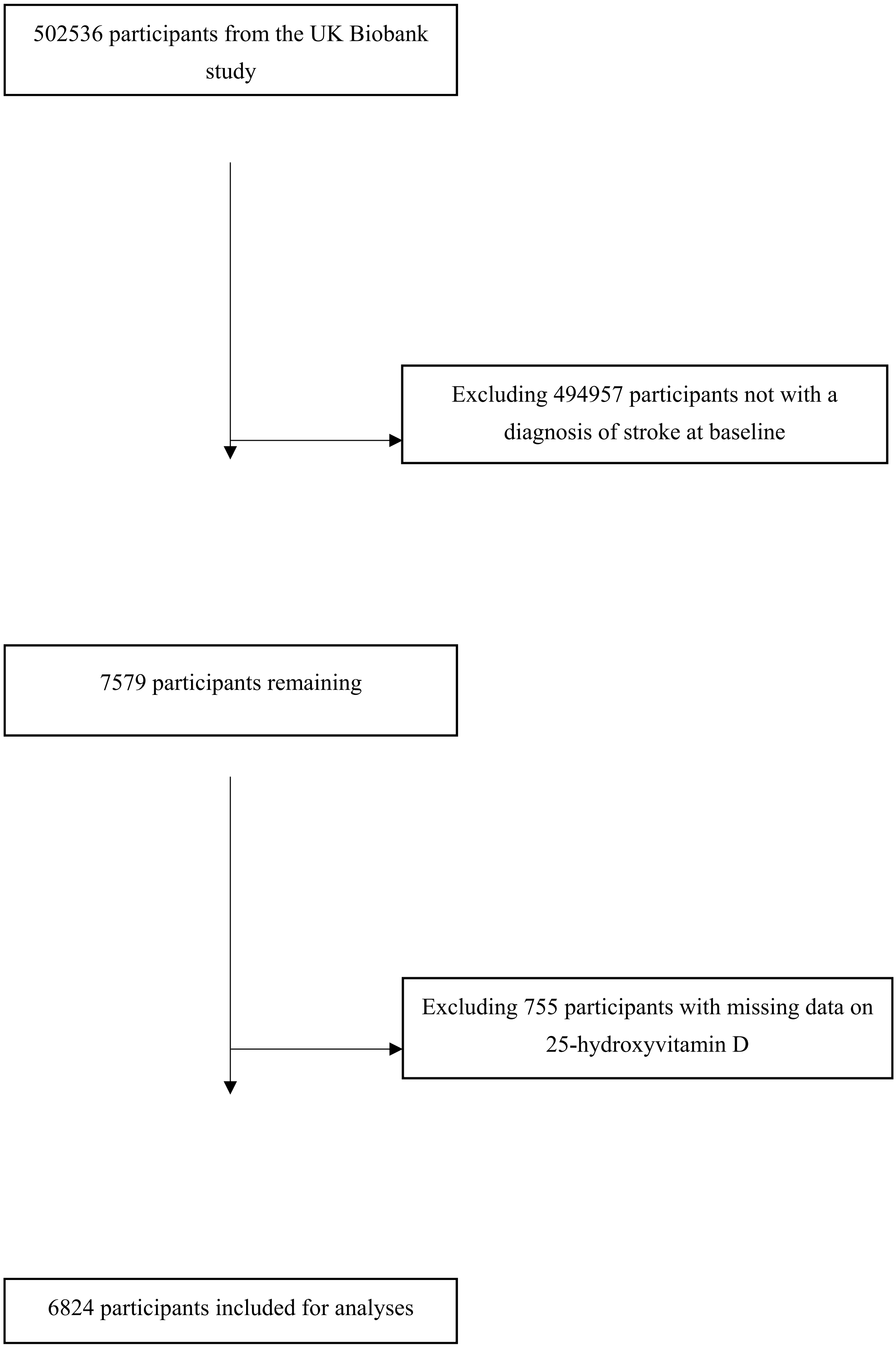

Supplemental Figure S2. Kaplan-Meier failure curve for incident recurrent stroke

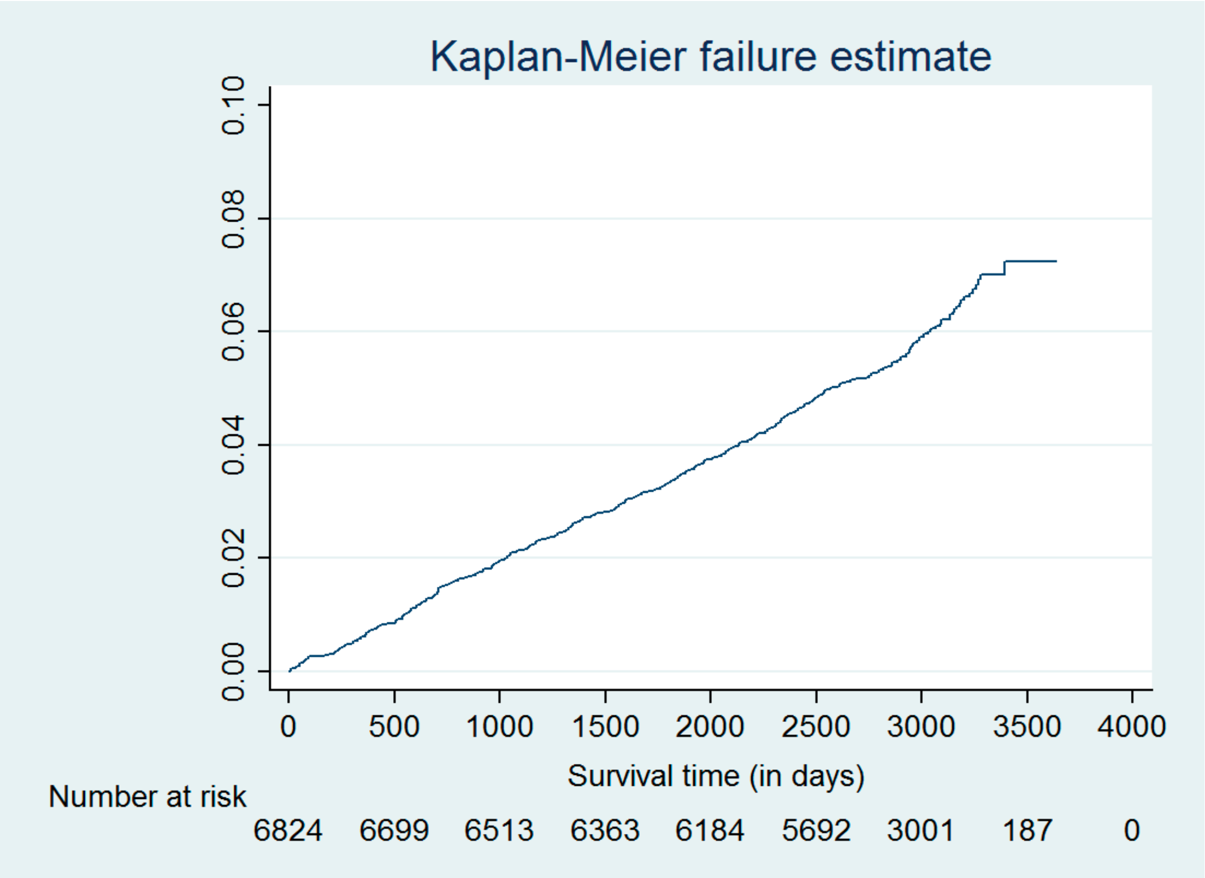

Supplemental Figure S3. Kernel density estimate for probability density function of 25(OH)D stratified by participants with and without recurrent stroke

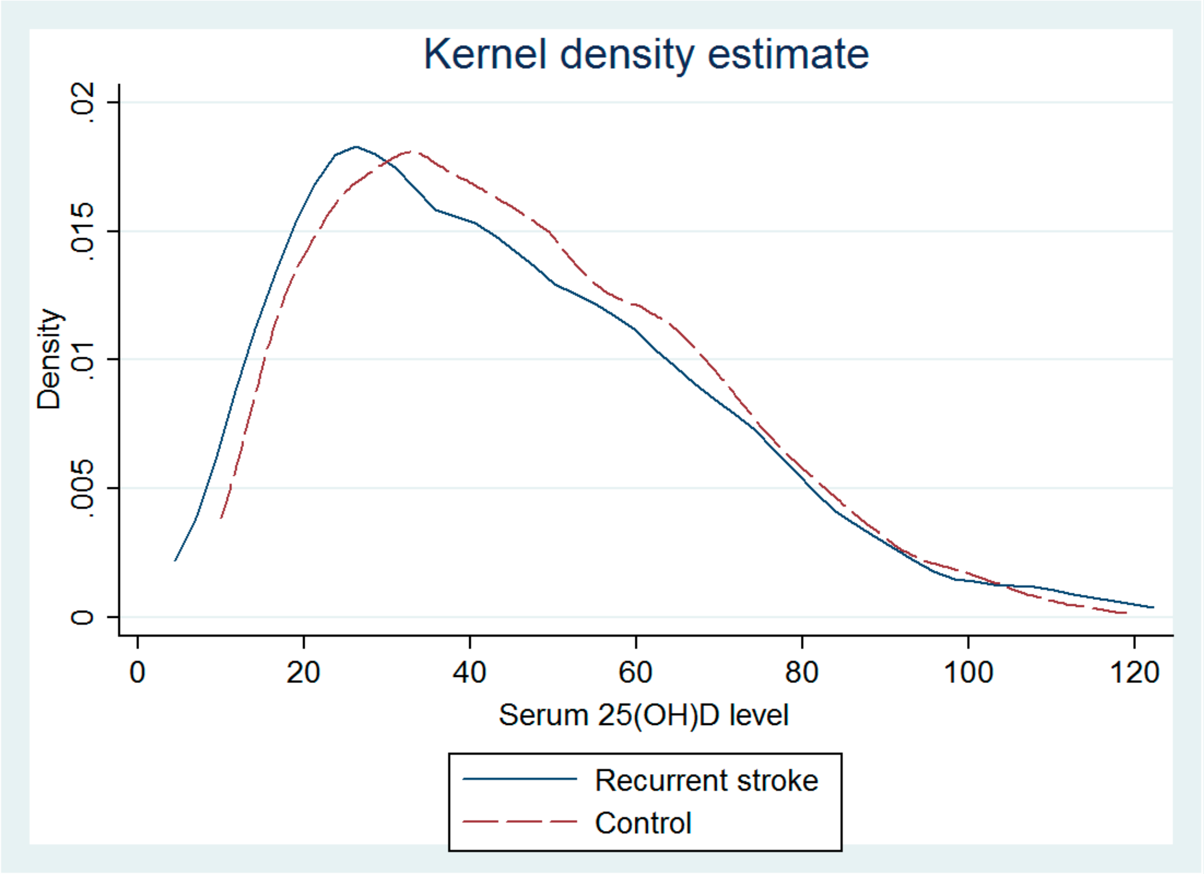

Supplemental Table S2. Sensitivity analysis results for the relationship between 25(OH)D and risk of recurrent ischemic stroke

Sensitivity analysis	25(OH)D level, in nmol/L[1]							
	10	20	30	40	50	60	70	80
**Competing risk analysis[1]**	Ref	1.40 (0.88 - 2.24)	0.69 (0.44 - 1.09)	0.76 (0.48 - 1.19)	0.70 (0.44 - 1.13)	0.66 (0.40 - 1.12)	0.82 (0.49 - 1.37)	0.70 (0.42 - 1.17)
**Model with further adjustment**								
Further adjusted for the month for 25(OH)D measures[2]	Ref	0.84 (0.64 - 1.11)	0.71 (0.44 - 1.16)	0.60 (0.35 - 1.04)	0.53 (0.32 - 0.87)	0.51 (0.31 - 0.82)	0.54 (0.34 - 0.86)	0.61 (0.38 - 0.99)
Further adjusted for the length between the stroke onset and blood sampling date[3]	Ref	0.84 (0.64 - 1.11)	0.71 (0.44 - 1.16)	0.60 (0.35 - 1.03)	0.53 (0.32 - 0.87)	0.51 (0.32 - 0.82)	0.55 (0.34 - 0.86)	0.62 (0.39 - 1.00)
Further adjusted for both the month and the length[4]	Ref	0.84 (0.64 - 1.10)	0.70 (0.43 - 1.14)	0.59 (0.34 - 1.02)	0.51 (0.31 - 0.85)	0.50 (0.31 - 0.80)	0.53 (0.33 - 0.85)	0.60 (0.37 - 0.97)

1 Results shown as subhazard ratios (95% confidence intervals) from the Fine and Gray models adjusted for age, sex, BMI, smoking and drinking, physical activity, comorbidities, medications and supplementation.

2 Results shown as hazard ratios (95% confidence intervals) from the Cox models that used restricted cubic splines and were adjusted for age, sex, BMI, smoking and drinking, physical activity, comorbidities, medications and supplementation, and the month for 25(OH)D measures.

3 Results shown as hazard ratios (95% confidence intervals) from the Cox models that used restricted cubic splines and were adjusted for age, sex, BMI, smoking and drinking, physical activity, comorbidities, medications and supplementation, and the length between stroke onset and blood sampling date.

4 Results shown as hazard ratios (95% confidence intervals) from the Cox models that used restricted cubic splines and were adjusted for age, sex, BMI, smoking and drinking, physical activity, comorbidities, medications and supplementation, the month for 25(OH)D measures and the length between stroke onset and blood sampling date.

Supplemental Figure S4. Restricted cubic splines showing 25(OH)D in relation to recurrent ischemic stroke with the lowest risk laying at 56.3 nmol/L

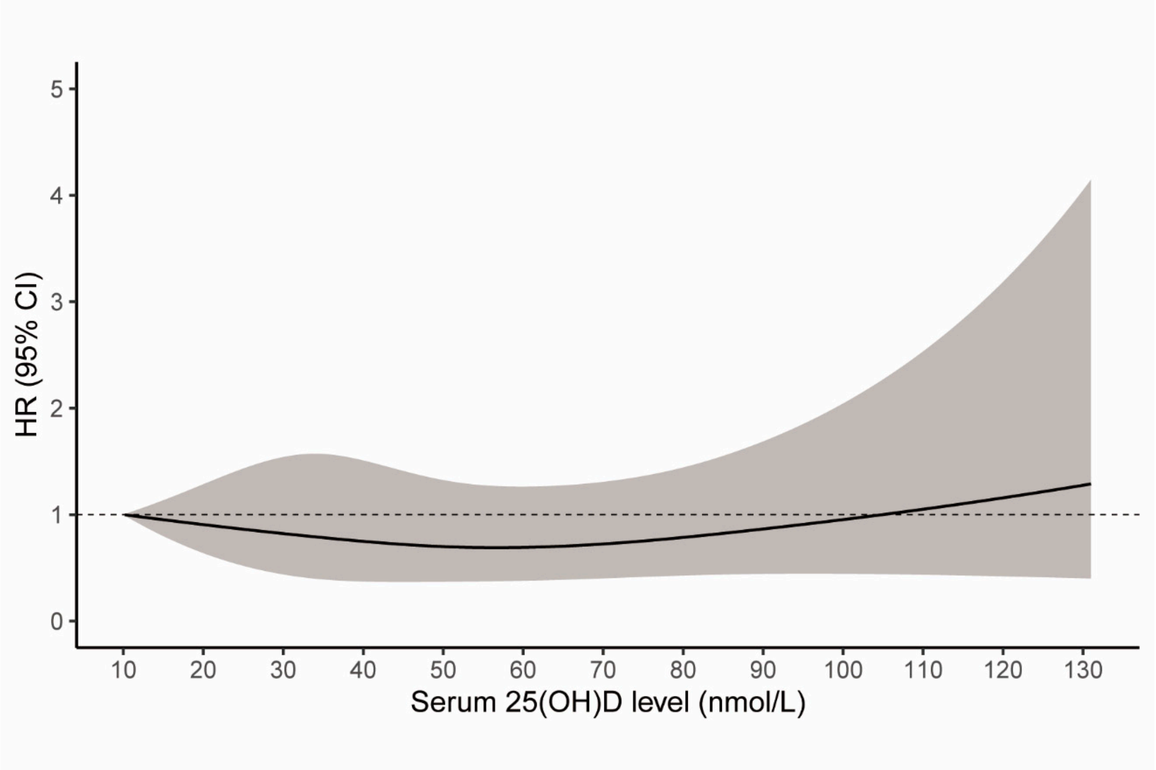

Supplemental Figure S5. Restricted cubic splines showing 25(OH)D in relation to recurrent hemorrhagic stroke with the potentially lowest risk laying at 41.2 nmol/L

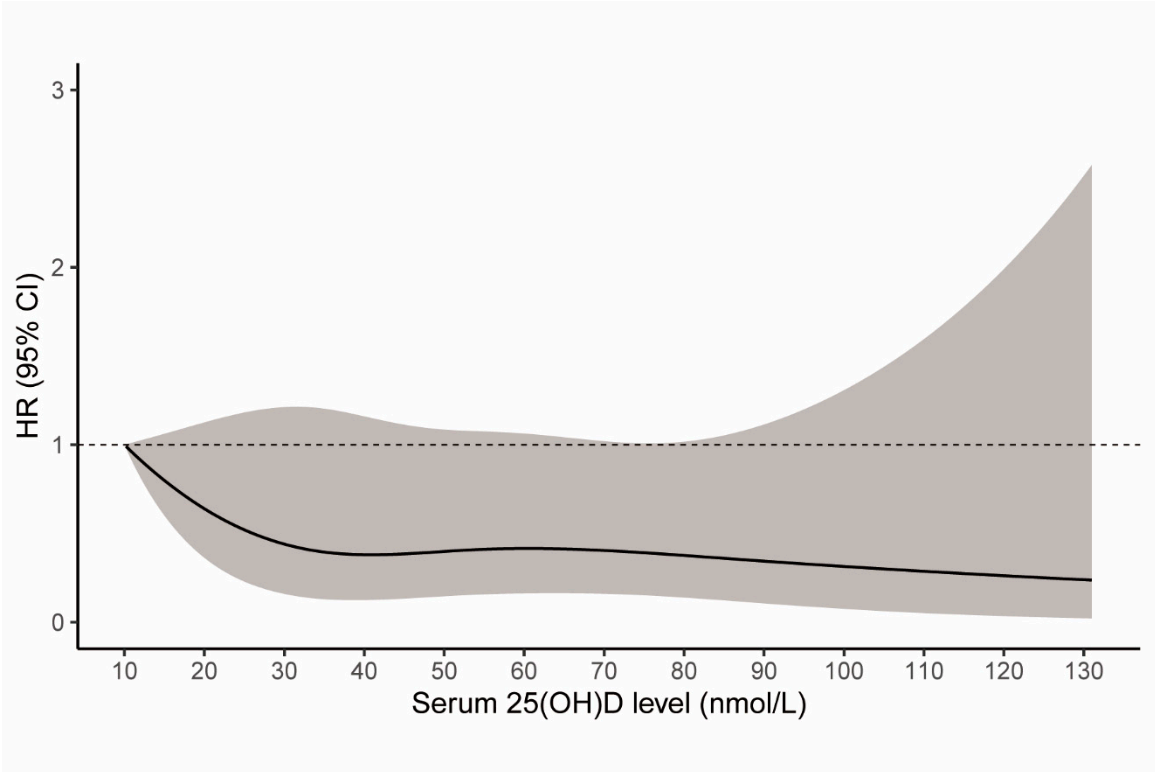

Supplemental Figure S6. Restricted cubic splines showing 25(OH)D in relation to recurrent stroke in males with the lowest risk laying at 58.7 nmol/L

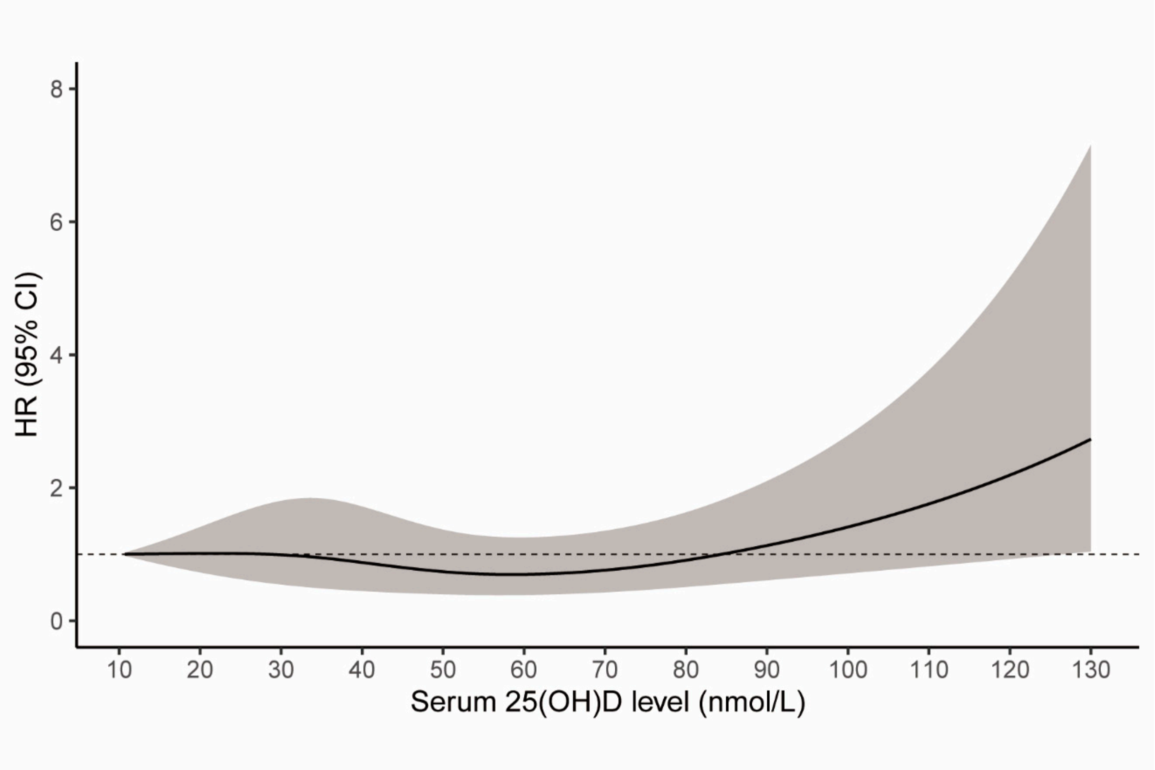

Supplemental Figure S7. Restricted cubic splines showing 25(OH)D in relation to recurrent stroke in females with the potentially lowest risk laying at 38.7 nmol/L

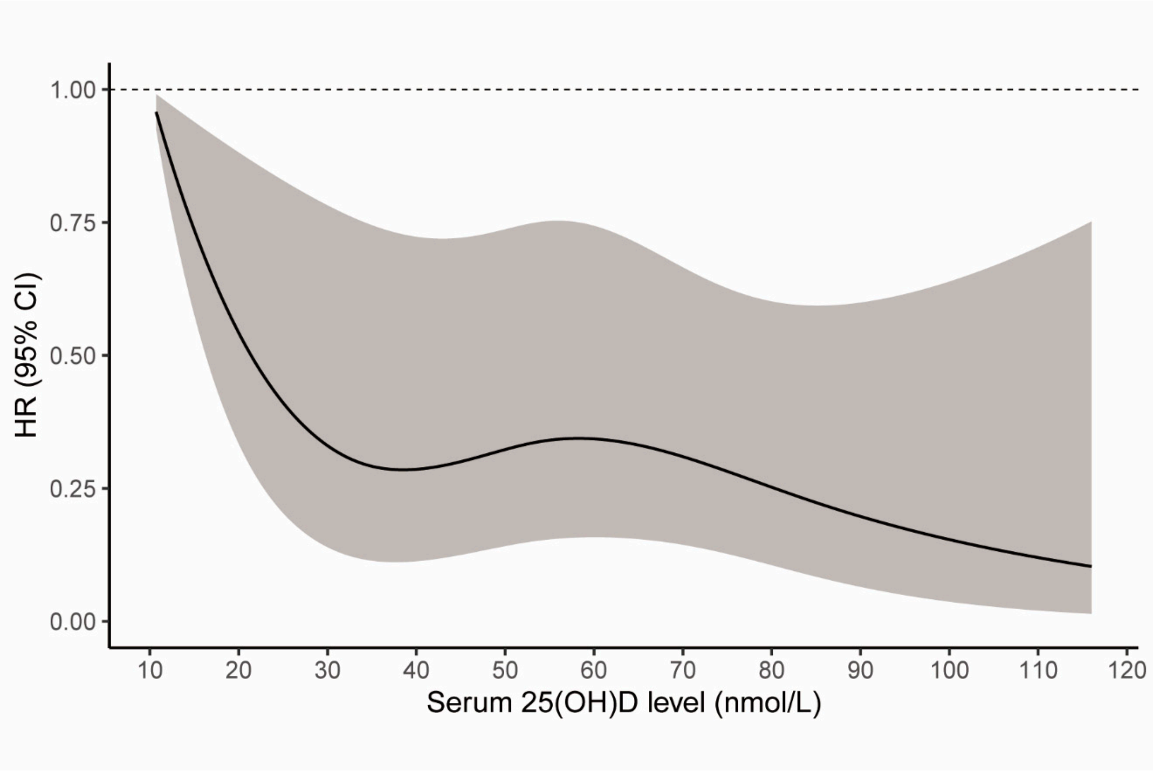

Supplemental Figure S8. Restricted cubic splines showing 25(OH)D in relation to recurrent stroke in patients less than 65 years with the lowest risk laying at 58.4 nmol/L

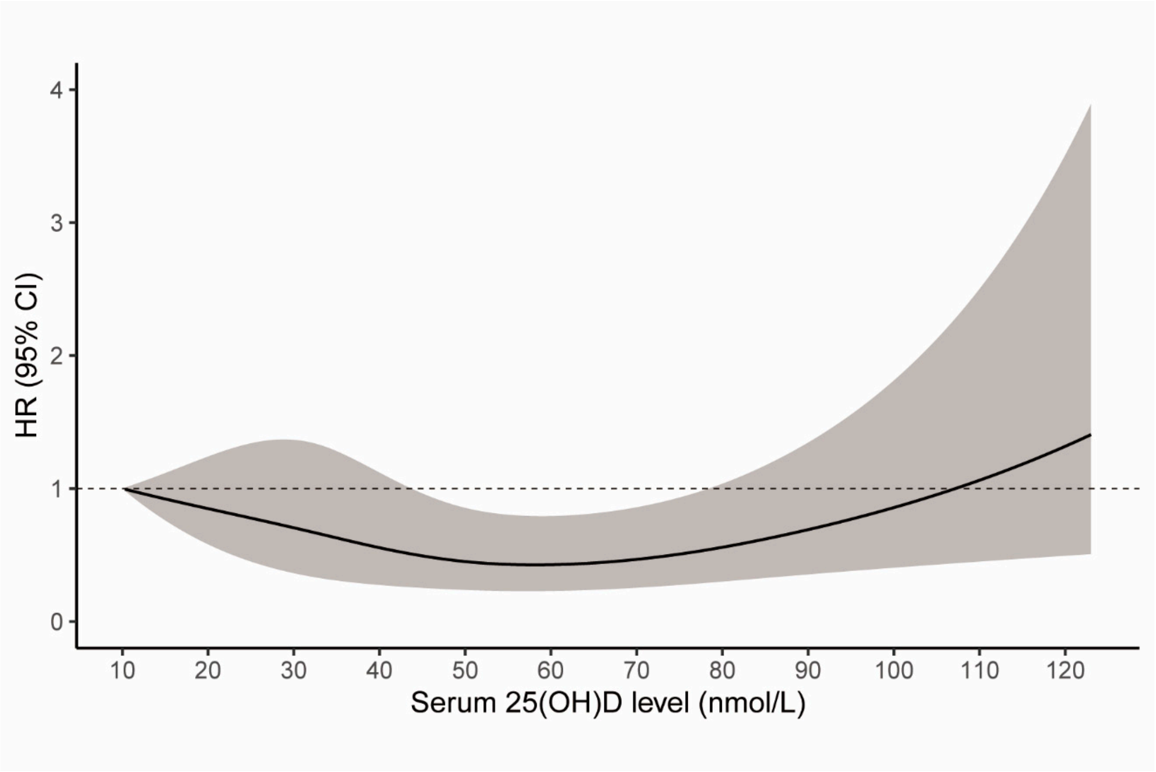

Supplemental Figure S9. Restricted cubic splines showing 25(OH)D in relation to recurrent stroke in patients no less than 65 years with the lowest risk laying at 53.3 nmol/L

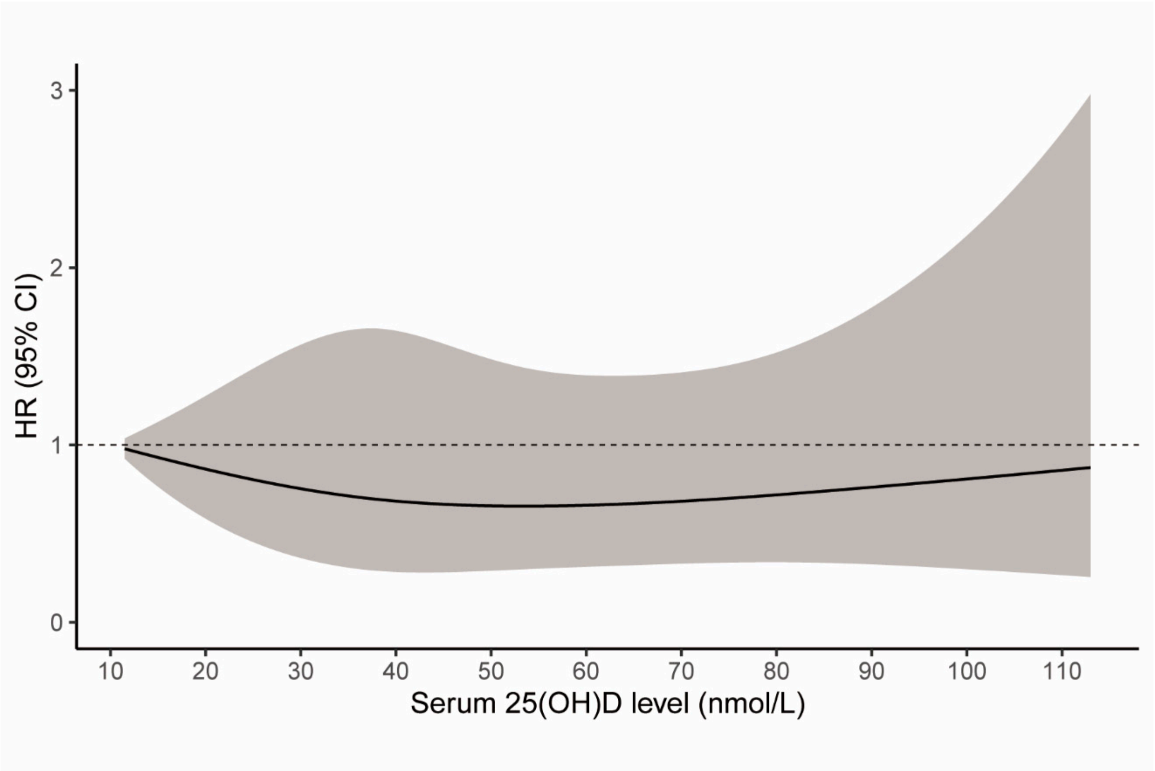

Supplement: Supplementary file 1 [file nutrients-14-01908-s001.zip › nutrients-1705970-supplementary.pdf]
